# Analysis of ecological quality changes and influencing factors in Xiangjiang River Basin

**DOI:** 10.1038/s41598-023-31453-7

**Published:** 2023-03-16

**Authors:** Yuanyuan Zhang, Liwen Yi, Binggeng Xie, Junhan Li, Jianyong Xiao, Jing Xie, Zhixing Liu

**Affiliations:** 1 College of Geographical Sciences, Hunan Normal University, Changsha, China; 2 Hunan Normal University Key Laboratory of Geospatial Big Data, Changsha, China; 3 Guangdong Guodi Planning Technology, Guangdong, China

**Keywords:** Ecosystem ecology, Ecosystem services

## Abstract

The Xiangjiang River Basin is an important part of the Yangtze River Basin and an important area in Hunan Province. Thus, taking steps to protect the ecological sustainability of the Xiangjiang River Basin, such as the construction of the protection of ecological security in Hunan Province and the Yangtze River Protection Law, is important for national projects However, research on the ecological quality of the Xiangjiang River Basin is mostly biased toward the evaluation of ecosystem services or an individual ecological index. Furthermore, a long-term evaluation of multiple indicators is lacking. Therefore, based on Google Earth Engine and geographic detectors, the remote sensing ecological index was used to evaluate this area. The year-by-year research on the Xiangjiang River Basin from 2001 to 2020 clarified its past ecological quality change trend, explored the reasons for the ecological quality change, and provided a basis for protecting its ecological quality. The following results are presented. (1) Regarding spatial distribution, areas with poor ecological environments are mainly distributed at the centers of Chang-Zhu-Tan, Hengyang, and various districts and counties. (2) Regarding the time variation, the ecological quality of the Xiangjiang River Basin from 2001 to 2020 showed a slight downward trend, with a downward slope of approximately − 0.0000357143; a rapid increase, with a growth rate of approximately 0.00395; And an overall improvement over 20 years. The areas with declining ecological quality are mainly located in the Chang-Zhu-Tan urban agglomeration, the city center of Hengyang, and the county centers of various county towns. (3) The factor detection results show that human factors play a key role in population density and land use, with average q values of 0.429 and 0.353, respectively. Among natural factors, elevation and slope play a key role, with average q values of 0.230 and 0.351, respectively; hence, Land use directly affect on the ecological quality in a location. These findings will provide important information for managers to formulate ecological restoration measures for the Xiangjiang River.

## Introduction

Recently, various research methods for ecological quality evaluation have emerged. Among these methods, the field monitoring method is deemed the most accurate method. However, the remote sensing evaluation method is relatively fast and convenient. It has high accuracy, which has contributed to its extensive use. Most evaluation methods are oriented to a single research object^1–5^ or artificially assigned weights^6–9^; these approaches heavily rely on the knowledge and experience of empowering people implementing them. The remote sensing ecological index (RSEI) proposed by Xu^10^, is considered a relatively scientific and objective ecological quality evaluation method. The advantage of this method is that it depends on the acquisition of four research objects, objects-surface greenness, temperature, dryness, and heat in the remote sensing images eliminating the need for data to study the ecological quality of large areas. Simultaneously, the four indicators are coupled using principal component analysis (PCA), which can avoid the interference caused by artificial weighting. Many studies have successfully applied this method in different areas, such as nature reserves^11,12^, mining areas^13,14^, soil erosion areas^15^, areas used in agricultural and animal husbandry^16^, and urban areas^17^.

The Xiangjiang River Basin is an important part of the Yangtze River Basin and an important area in Hunan Province. This river is a key area for biodiversity conservation and environmental and ecological improvement in Hunan Province. The core areas of the economic and social development of Hunan Province include a concentrated population, a developed economy, convenient transportation, and a rich cultural heritage. Hence, the traditional extensive development model has not fundamentally changed. However,the problems of watershed resources and the ecological environment have become increasingly prominentrecently,and the pressure for sustainable development is rising. Furthermore, changes in its ecological environment significantly impact the environmental protection and ecological security of the Dongting Lake Basin and surrounding provinces. Thus, protecting the Xiangjiang River Basin is crucial to protecting the ecology of the Yangtze River Basin and Hunan Province, and ecological security and stability play irreplaceable roles in this task.

From 2000 to 2010, the scale of Urban area expanded unprecedentedly as the Yangtze River Basin entered the golden period of economic development^18^, Recently, with the further growth of the economy and population and various human events, including the mining and reclamation of the ecological environment,parts of the Xiangjiang River Basin have continued to worsen because of the influence of several factors. This development severely affects the ecological environment security of the basin. For example, Hunan Province is famous as the home of nonferrous metals, and several studies have shown that most nonferrous metal minerals in Hunan Province are concentrated in the Xiangjiang River Basin^19^, However, mining has caused a series of ecological problems, such as soil erosion.Hence, the Hunan Provincial People's Government has implemented the “Overall Plan for the Scientific Development of the Xiangjiang River Basin”, which included agricultural nonpoint source pollution and Comprehensive control of agricultural pollution sources, returning farmland to forests and wetlands, standardizing industrial and agricultural production, and strengthening the construction of natural reserves to improve the ecological quality of the Xiangjiang River Basin effectively. The ecological quality of the Xiangjiang River Basin has been significantly improved through the implementation of these actions, but the threat to ecological quality is still severe. Therefore, it is urgent to study the changes in the ecological quality of the Xiangjiang River Basin and the factors influencing them.

Majority studies on ecological quality in Hunan Province or the Xiangjiang River Basin relate to ecosystem services^20–24^. For example, Wang al.^23^ used land use types to study the supply and demand relationship of ecosystem services. Xiong et al.^25^ studied the impact of land use changes on the temporal and spatial evolution of ecosystem service values. Furthermore,Xue et al.^22^ used the equivalent factor method to investigate the ecosystem service value of rice fields in Hunan Province, and eight aspects including water conservation were discussed. Nevertheless,research on the impact of surface temperature and buildings on ecological quality is scarce, and these two factors are crucial in ecology. Moreover, Previous studies have rarely involved long time series studies, and the selection of influencing factors was relatively single. Therefore, this paper further refines the study on the Dongting Lake basin^26^ to investigate the factors that influenced the changes in the ecological quality of the Xiangjiang River Basin over a long time series.

Traditional research methods usually screen, download, and process remote sensing images, then calculate PCA weights for analysis, resulting in a large workload, and complications in achieving the efficient performance of long time series research. Moreover,only one or two images are selected for research each year, increasing the probability of errors in the image selection process. Google Earth Engine (GEE) can access numerous public resources to process remarkably large geospatial datasets, which is particularly suitable for large-scale and long time series monitoring. This demonstrates the potential application of GEE in ecological and environmental quality evaluation as well as in the detection of variations that indicate natural disasters.Zhang et al.^27^ successfully used GEE to study the city of Xi’an, China.

Therefore, this paper uses the GEE online processing platform to further study the ecological quality of Hunan Province and explore the factors influencing long-term ecological quality changes and spatial differentiation in Hunan Province. Taking the Xiangjiang River Basin in Hunan Province as the research area, the RSEI in the study area is calculated, and the spatial and temporal changes in ecological quality from 2001 to 2020 are explored. The coefficient of variation (CV), Hurst index, and Sen-MK trend test methods were used. Furthermore, the temporal and spatial variation trends of ecological quality from 2001 to 2020 were used. Finally, geographic detectors were used to explore the influencing factors of the temporal and spatial variation characteristics of ecological quality in the Xiangjiang River Basin from 2001 to 2020.

## Materials and methods

### Study area

**Figure 1:**
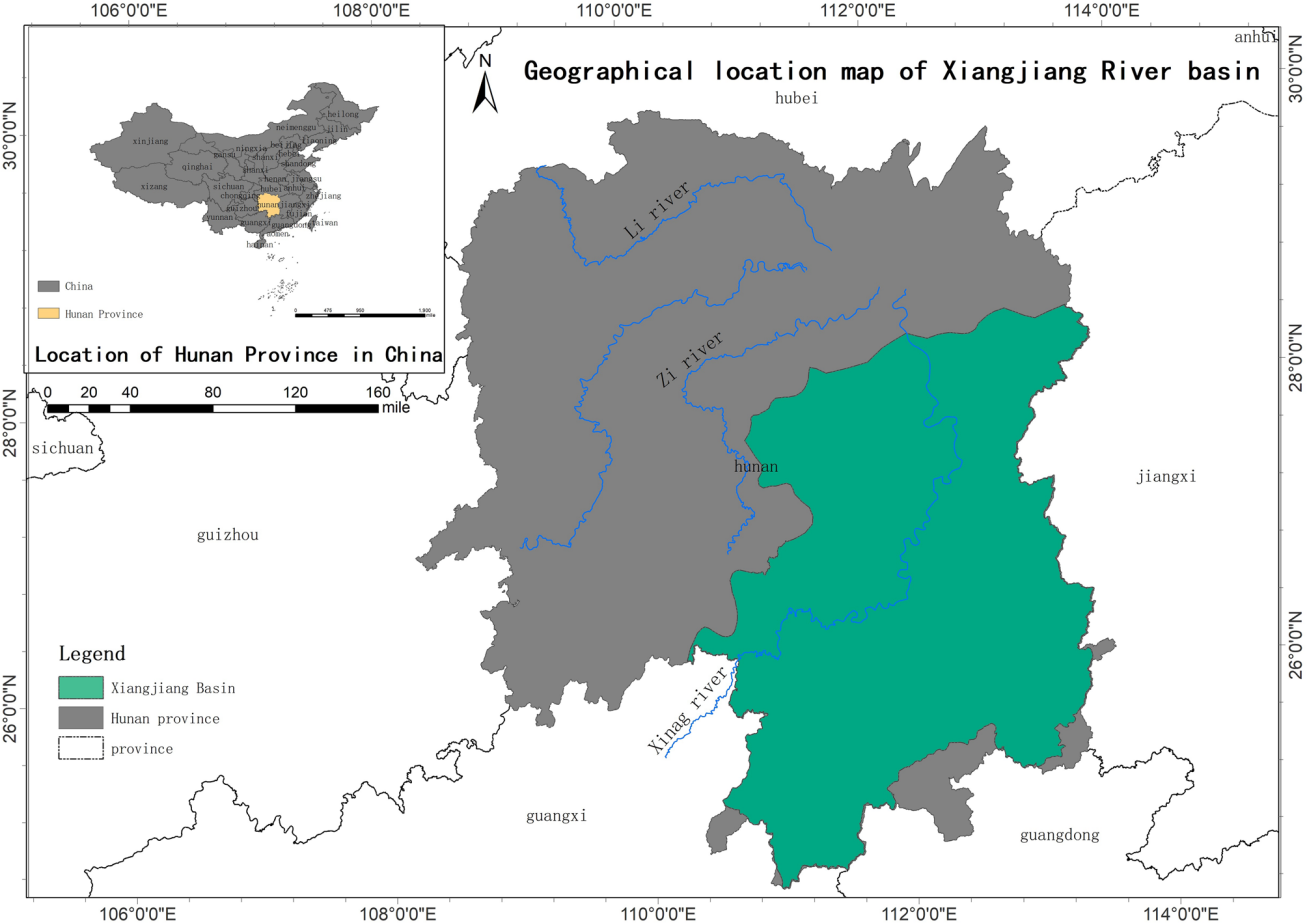
Location map of hunan section of the Xiangjiang River Basin.

The Xiangjiang River Basin is located in south of the Jingjiang River in the middle reaches of the Yangtze River and in north of the Nanling Mountains. The water system in the basin has many tributaries, and the total area of the basin reaches 96,567 km^2^. The watershed area in Hunan Province is 84,707 km^2^, constituting for 88.90% of the total watershed area. The river basin is densely populated with towns, making it the most economically developed area in Hunan Province. By the end of 2015, the GDP of the Xiangjiang River Basin had reached 2.124858 trillion yuan, and the total population had reached 39.3855 million, accounting for 73.15% and 58.10% of the GDP and the total population of Hunan Province, respectively. The accelerated expansion of urban construction land, the unprecedented increase in the breadth and intensity of human activities, intensification of environmental pollution caused by urban development, and unprecedented pressure on the ecological environment can be attributed to the acceleration of urbanization and industrialization (Fig. 1).

### Data resource

Previous studies used Landsat images in their researches^28–30^. However, a single year of Landsat images cannot cover the entire Xiangjiang River because of the large area of the Xiangjiang River Basin and the need for remote sensing images of the vegetation growth season from June to September. Therefore, temporal accuracy must be sacrificed to obtain high spatial resolution. The current study investigates the long-term ecological quality of the Xiangjiang River Basin. Thus, MODIS images with a relatively high temporal resolution were selected to achieve a high level of temporal resolution in the study area for annual monitoring. Simultaneously, except for the constant data during the year, the rest of the data were selected from the average data of the vegetation growth season in the southern region, that is, the average data from June to September (the natural conditions are relatively similar), to ensure the similarity and comparability of ecological results. In addition, the random error caused by the image can contribute to the similarity in the weights of the four indicators in different years. The data sources are shown as Table 1.

**Table 1:** Data source and description.

Data	Data source	Usage	Resolution (m)	Remark
MOD09A1	https://earthengine.google.com/	MNDWI (modified normalized difference water index), WET, NDBSI (normalized differential building-soil index)	500	Cloud cover below 5%
MOD11A2	https://earthengine.google.com/	LST	1000	
MOD13A1	https://earthengine.google.com/	NDVI (normalized difference vegetation index)	500	
DEM	https://urs.earthdata.nasa.gov	Elevation, slope	12.5	
CLCD	“30 m annual land cover and its dynamics in China from 1990 to 2019”^31^	Land use	1000	Contains 2020 data
GDP	China County Statistical Yearbook	GDP	100	
Precipitation	National Climatic Data Center	Precipitation	1000	
Population density	World pop	Population density	1000	
Downtown data	National Bureau of Statistics of China	Distance from the city center	1000	

### Research methods

#### Research methods of for remote-sensing-based ecological index

The ecosystem is an intricate system with a high degree of complexity. Among the many natural factors that reflect ecological quality, the RSEI model has four selected indicators closely related to human survival: greenness, humidity, heat, and dryness^10^. The four indicators are also human’s intuitive sense of ecology quality. Therefore, scholars in the same field often use them to evaluate ecosystems^28–30,32^. In previous studies, many scholars have revised the RSEI and added different indicators, such as sand degree^33^ and aerosol optical depth^27^. However, Xu^34^ believed that these newly added indicators belong to the impact factors of ecological quality, whereas the nonecological factors themselves. Therefore, the initial four factors originally proposed should be used for the RSEI calculation. Moreover, more impact factors can be selected in the subsequent driving force analysis. Based on remote sensing technology, the information from the four indicators is extracted from remote sensing images using thematic information enhancement technology. The tasseled-cap-transformed humidity component represents moisture, NDVI represents greenness, the surface temperature represents heat, and the building and bare soil indexes represent dryness. Thus, RSEI can be expressed as follows:

1 $$ {\text{RSEI }} = f(Wet,NDVI,NDBSI,LST) $$

Here, NDVI, WET, LST, and NDBSI indicata greenness, wetness, heat and dryness, respectively. If the load signs of ecologically positive indicators have negative signs,then 1 − f(*Wet*, *NDVI*, *NDBSI*, *LST*) processing is required. The NDVI and WET load signs were positive in this study. Therefore, the operation is unnecessary^34^.

- NDVI

The normalized difference vegetation index (NDVI) represents surface vegetation characteristics^35^. The calculation formula for NVDI is as follows:

2 $$ {\text{NDVI }} = \, \left( { \, \rho {\text{NIR }}{-} \, \rho {\text{RED }}} \right)/\left( { \, \rho {\text{NIR }} + \, \rho {\text{RED }}} \right). $$

Here, ρNIR and ρRED are the near-infrared and red bands of MOD13A1, respectively.

(b) WET

Soil can be used to monitor land degradation and is an important factor in studying ecological and environmental changes. Therefore, the MOD09A1 surface reflectance product based on the modified MODIS tasseled cap transformation formula^36^ is used to calculate the humidity index in the study area. The calculation formula is as follows:

3 $$ {\text{WET }} = {\text{ A1}}\rho {1 } + {\text{ A2}}\rho {2 } + {\text{ A3}}\rho {3 } + {\text{ A4}}\rho {4 } + {\text{ A5}}\rho {5 } + {\text{ A6}}\rho {6 } + {\text{ A7}}\rho {7}{\text{.}} $$

Here, A1 to A7 are 0.1147, 0.2489, 0.2408, 0.3132, − 0.3112, − 0.6416 and − 0.5087; ρi represents the surface reflectivity of i bands of MOD09A1.

(c) NDBSI

Studies have shown that urban expansion affects the region’s water, soil, atmosphere, and biodiversity^37^. A large area of bare land indicates severe land degradation. In this paper, the average of the building index (IBI)^38^ and the bare soil index (SI)^39^ will be used to represent NDBSI. The calculation formula of NDBSI is as follows:

4 $$\mathrm{NDBSI}=\frac{\mathrm{IBI}+\mathrm{SI}}{2},$$

5 $$\mathrm{IBI}=\frac{\frac{2\mathrm{\rho SWIR}1}{\mathrm{\rho SWIR}1+\mathrm{\rho NIR}}-[\frac{\mathrm{\rho NIR}}{\mathrm{\rho NIR}+\mathrm{\rho RED}}+\frac{\mathrm{\rho GREEN}}{\mathrm{\rho GREEN}+\mathrm{\rho SWIR}1}]}{\frac{2\mathrm{\rho SWIR}1}{\mathrm{\rho SWIR}1+\mathrm{\rho NIR}}+[\frac{\mathrm{\rho NIR}}{\mathrm{\rho NIR}+\mathrm{\rho RED}}+\frac{\mathrm{\rho GREEN}}{\mathrm{\rho GREEN}+\mathrm{\rho SWIR}1}]},$$

6 $$\mathrm{SI}=\frac{(\mathrm{\rho SWIR}1+\mathrm{\rho RED})-(\mathrm{\rho Blue}+\mathrm{\rho NIR})}{(\mathrm{\rho SWIR}1+\mathrm{\rho RED})+(\mathrm{\rho Blue}+\mathrm{\rho NIR})}.$$

Here, ρi represents the different bands of image MOD09A1.

(d) LST

Temperature and humidity are important ecological factors that drive changes in the ecological environment^40^. MODIS surface temperature image data were used and converted to Celsius for calculation^32^. The specific formula is as follows:

7 $$ {\text{LST }} = \, 0.0{2} \times {\text{DN}}_{{\text{S}}} - {273}.{15}{\text{.}} $$

Here, DNS represents the gray value of MOD11A2.

(e) Body of water mask

The humidity index represents only soil and vegetation moisture but cannot express water body humidity. Thus, the modified normalized difference water index (MNDWI) was used to mask the water body to increase the accuracy of the ecological environment evaluation. Its calculation formula is as follows:

8 $$ {\text{MNDWI }} = \, \left( {{\text{Green}} - {\text{MIR}}} \right)/\left( {{\text{ Green }} + {\text{ MIR}}} \right). $$

Here, Green is the green light band data in MODIS remote sensing data, and MIR is the mid-infrared band data^41^.

The MODIS images in the study area were screened through the Google Earth online platform, and relevant cloud removal calculations were performed. Simultaneously, the threshold of the MNWDI was adjusted to increase its suitability for the selected remote sensing images. After that, the calculation formula from the Google Earth online platform was used to calculate the four indicators. Next, the four indicators were standardized, and the PCA algorithm from the Google Earth online platform was used to perform PCA on the four indicators after standardization. Finally, the analysis was exported to ArcGIS software for subsequent operations.

#### Eco-environmental assessment methods

1. The coefficient of variation can be unrestricted by the mean value. This paper used the coefficient of variation to study the ecological stability of our study area over the years, and the equation is as follows^42^:
  9 $$ {\text{C}}_{{\text{V}}} = \, \sigma / \mu $$

Here: σ and μ denote the standard deviation and mean of the RSEI time series, respectively. A large CV value leads to large fluctuations in the RSEI time series and drastic changes. Conversely, a small CV value leads to minimal fluctuations in the RSEI time series and a stable state.

(2) The mode of change analysis used a combination of the Sen slope estimation of the median (Theil–Sen) and MK test to determine the trend of RSEI in the study area, and the calculation formula is as follows^43^:
  10 $$ \beta = {\text{ median}}\left( {\frac{RSEIj - RSEIi}{{j - i}}} \right),{\text{ i}} < {\text{ j}}{.} $$

Here: β is the median trend of Theil–Sen. RSEIj and RSEIi denote the RSEI values of each image element in Years j and i, respectively. If β > 0, then the ecological environment quality of the region demonstrates an improvement trend. Otherwise, it is in a worsening trend. The MK test is a nonparametric statistical test with the advantage that the data need not obey a normal distribution and remain unaffected by outliers. The calculation method is as follows:

11 $$\mathrm{S}=\sum_{i=1}^{n-1}{\sum }_{j=i+1}^{n}sgn(RSEIj-RSEIi)$$

Here RSEIi and RSEIj are pairwise values and sgn is the sign function, calculated as follows:

12 $$\mathrm{Sgn}(\mathrm{RSEIj}-\mathrm{RSEIi})=\left\{\begin{array}{c}1,RSEIj-RSEIi>0\\ 0,RSEIj-RSEIi=0\\ -1,RSEIj-RSEIi<0\end{array}\right.$$

The trend test was performed using the Statistic Z. The Z value was calculated as follows:

13 $$\mathrm{Z}=\left\{\begin{array}{c}\frac{S}{\sqrt{var\left(S\right)}},S>0\\ 0, S=0\\ \frac{S+1}{\sqrt{var\left(S\right)}},S>0\end{array}\right.$$

Here, var(S) denotes the variance of S. The results are calculated as follows:

14 $$\mathrm{Var}(\mathrm{S})=\frac{n(n-1)(2n+5)}{18}$$

Theil–Sen Median slope estimation was conducted separately using MATLAB software, while the MK significance test was performed to find the critical value Z_1−α/2_ in the normal distribution table at the given significance level. When |Z|≤ Z_1−α/2_, the original hypothesis was accepted (the trend was not significant); if |Z|> Z_1−α/2_, then the original hypothesis was rejected (the trend is considered significant). In this paper, given that the significance level α = 0.05, the critical value of Z_1−α/2_ =  ± 1.96, when the absolute value of Z is greater than 1.96, the trend passes the significance test with 95% confidence. The multiyear change of RSEI is more significant; otherwise, it is not significant. Furthermore, the results of the Sen slope estimation and MK test are combined to obtain five types of multiyear RSEI changes in the Xiangjiang River basin: severe degradation, slight degradation, no change, slight improvement, and significant improvement. The graded results are shown in Table 2.

**Table 2:** Theil–Sen Median-Mann–Kendall trend test category table.

β	Z	Trend characteristics
β > 0	Z > 1.96	Significant degradation
	Z < 1.96	Slight degradation
β = 0	Z	No change
β < 0	Z < 1.96	Slight improvement
	Z > 1.96	Significant improvement

- The Hurst index, proposed by the British hydrologist Hurst, can be used to describe the sustainability of time series data quantitatively. The Hurst index is calculated in this paper based on the R/S method, and the specific calculation principle is as follows^42^.
- Define the difference sequence {ξ(t)}.
  15 $$ \xi \left( {\text{t}} \right) \, = {\text{ RSEI}}\left( {\text{t}} \right) - {\text{RSEI}}\left( {{\text{t}} - {1}} \right),{\text{t }} = { 1},{2}, \ldots ,{\text{n}}{.} $$

Here: RSEI(t) is the image element value of RSEI at moment t.

(b) Define the mean sequence.
  16 $$<\upxi >\mathrm{t }=\frac{1}{\uptau }\sum_{1}^{\uptau }\xi (t),\uptau =\mathrm{ 1,2},3\dots \mathrm{n}$$
(c) Define the mean sequence.
  17 $$\mathrm{X}(\mathrm{t},\uptau ) ={\sum }_{1}^{\uptau }(\xi \left(t\right) - <\xi >t),1 \le \mathrm{ t }\le\uptau $$
  ① Define the extreme deviation.
    18 $$ {\text{R}}(\tau ) \, = {\text{ max}}({1} \le {\text{t}} \le \, \tau ){\text{X}}({\text{t}},\tau ) - {\text{min}}({1} \le {\text{t}} \le \, \tau ){\text{X}}({\text{t}},\tau ) $$
  ② Define the standard deviation series.
    19 $$ {\text{R}}\left( \tau \right) \, = {\text{ max(1}} \le {\text{t}} \le \tau ){\text{X}}\left( {{\text{t}},\tau } \right) - {\text{min(1}} \le {\text{t}} \le \tau ){\text{X}}\left( {{\text{t}},\tau } \right) $$
  ③ Calculate the Hurst exponent.
    20 $$ {\text{R}}\left( \tau \right)/{\text{S}}\left( \tau \right) \, = \, \left( {{\text{c}}\tau } \right){\text{H}} $$

The value of H, the Hurst index, is obtained by least squares fitting with the following formula.

21 $$ {\text{log}}\left( {{\text{R}}/{\text{S}}} \right){\text{n }} = {\text{ a}} + {\text{H}} \times {\text{log}}\left( {\text{n}} \right) $$

The above studies were conducted in MATLAB.

#### Driving force research method

Relevant studies have shown^44^ that land use types can directly affect ecological quality. Rainfall is an important climate-influencing factor, and population and socioeconomic development (population density and GDP) are crucial human disturbance factors^45,46^. Jiang et al.^47^ and Jia et al.^46^ indicated that a large population density, indicates a highly negative impact on ecological health. Moreover, since rainfall is an important climate influencing factor, elevation and slope are the basis of the natural environment, and the distance from the city center, to a degree, can reflect the economic development of a region. Therefore, this paper selects the distance from the city center (X1), GDP (X2), land use (X3), precipitation (X4), population density (X5), elevation (X6) and slope (X7) of seven independent variable factors to explore the factors influencing temporal and spatial variations of RSEI. According to the spatial differentiation of RSEI, precipitation data, population density data, elevation, slope, and data on distance from the city center were divided into five categories according to the natural breakpoint method, land use methods are divided into four categories except for waters, according to the land use classification data of the Chinese Academy of Sciences, and the GDP data were classified according to counties. Furthermore, considering the computational upper limit of EXCEL, a 2 × 2 km grid was used to sample the independent variables. The geographic detector EXCEL plugin developed by Wang Jinfeng was used to perform factor and interactive detections of the seven influencing factors. The geographic detector was used to check the results of factor detection during the calculation. If P < 0.05, then the result of factor detection is realistic. The mathematical principles of the factor and interaction detections of geodetectors can be explained following Wang’s paper^48^.

## Results

### Temporal and spatial characteristics of ecological quality

Table 3 shows the PCA results of the RSEI. (1) The PCA1 contribution rate between 2001 and 2020 was higher than 70% in most years during those decades. (2) The contribution rate of the four indicators in PCA1 shows that the average values of the multiyear eigenvectors of NDVI, LST, WET, and NDBSI are 0.448, − 0.568, 0.258, and − 0.632, respectively. Among the four indicators, the positive indicator is the greenness index (NDVI) and humidity index (WET) and the negative indicators are the dryness index (NDBSI) and heat index (LST). This shows that greenness and humidity have a positive effect on the ecological environment, while dryness and temperature have a positive effect on the ecological environment. Furthermore, a negative impact, which corresponds with the actual situation and previous research, was observed^49–51^; Following eigenvectors, the effects of the four indicators on ecology were relatively small. RSEI is affected by negative indicators, and the humidity index plays the weakest role, indicating the crucial impact of human activities on the ecological environment. (3) The positive and negative signs of other principal components (PC2, PC3 and PC4) are unstable, hard to indicate the ecological phenomenon. PC1 integrates the characteristic information of the four indicators and is consistent with the actual situation. Therefore, the RSEI was formed by integrating the four indicators through PC1.

**Table 3:** Analysis results of RSEI principal component from 2001 to 2020.

Index	NDVI	LST	WET	NDBSI	EV(PC1)	ECR (PC1%)
2001	0.434	− 0.656	0.217	− 0.576	0.03	67.3
2002	0.429	− 0.554	0.255	− 0.665	0.02	56.8
2003	0.423	− 0.646	0.269	− 0.574	0.03	76.1
2004	0.464	− 0.622	0.294	− 0.557	0.03	70.7
2005	0.311	− 0.645	0.371	− 0.596	0.03	66
2006	0.378	− 0.597	0.294	− 0.643	0.03	63.7
2007	0.394	− 0.512	0.308	− 0.697	0.03	72.6
2008	0.391	− 0.642	0.296	− 0.589	0.03	63.9
2009	0.453	− 0.568	0.251	− 0.639	0.03	71.3
2010	0.433	− 0.566	0.262	− 0.652	0.03	70.5
2011	0.464	− 0.553	0.214	− 0.657	0.03	73.2
2012	0.465	− 0.602	0.225	− 0.607	0.03	67.9
2013	0.461	− 0.618	0.219	− 0.597	0.03	75.1
2014	0.501	− 0.545	0.189	− 0.644	0.03	66.7
2015	0.522	− 0.408	0.229	− 0.712	0.03	67.1
2016	0.507	− 0.532	0.207	− 0.644	0.03	73.6
2017	0.477	− 0.581	0.235	− 0.615	0.03	74.1
2018	0.507	− 0.516	0.256	− 0.641	0.03	76.5
2019	0.487	− 0.502	0.261	− 0.664	0.03	75
2020	0.435	− 0.571	0.259	− 0.645	0.03	71.2

**Figure 2:**
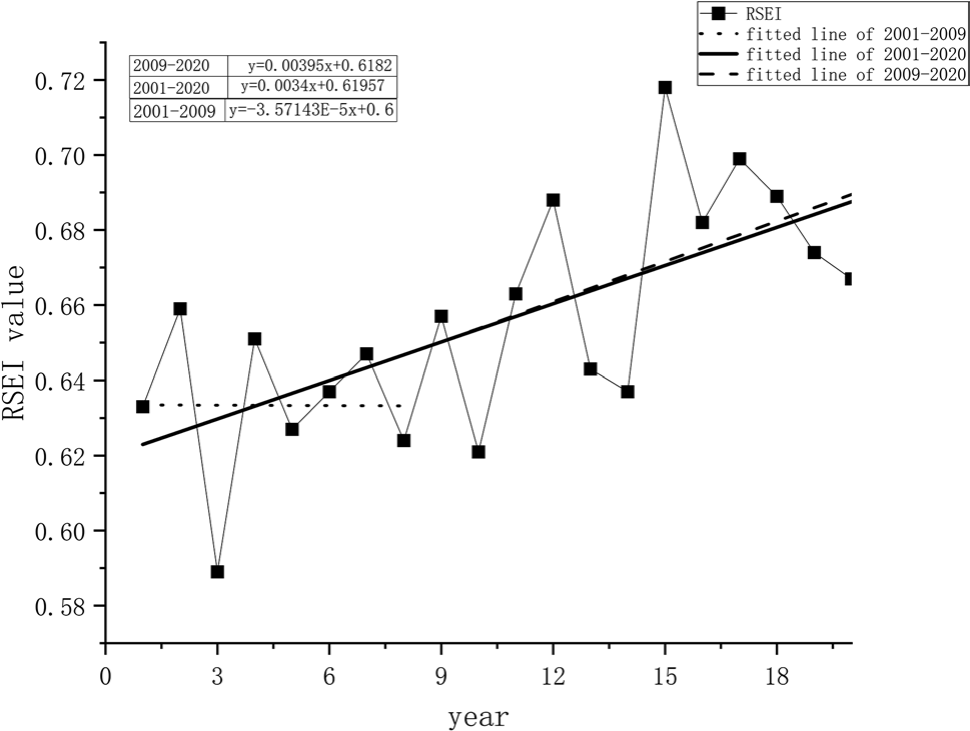
The interannual trend of the mean value of the RSEI.

Following statistics, the change in the trend of RSEI from 2001 to 2020 is shown in Fig. 2, where the maximum value appeared in 2015 (0.718), and the minimum value appeared in 2003 (0.589), demonstrating an overall value between 0.6 and 0.8 In the past 20 years, the overall ecological quality has been upward, and the upward slope is approximately 0.0034 (p < 0.05). Before 2009, the ecological quality had a slight downward trend, and the absolute value of the slope was small at only − 0.0000357143 (p < 0.05). However, quality began to rise rapidly after 2009. The slope is approximately 0.0039 (p < 0.05), but the ecological quality has been declining again in recent years as observed from the line chart (Fig. 2).

**Figure 3:**
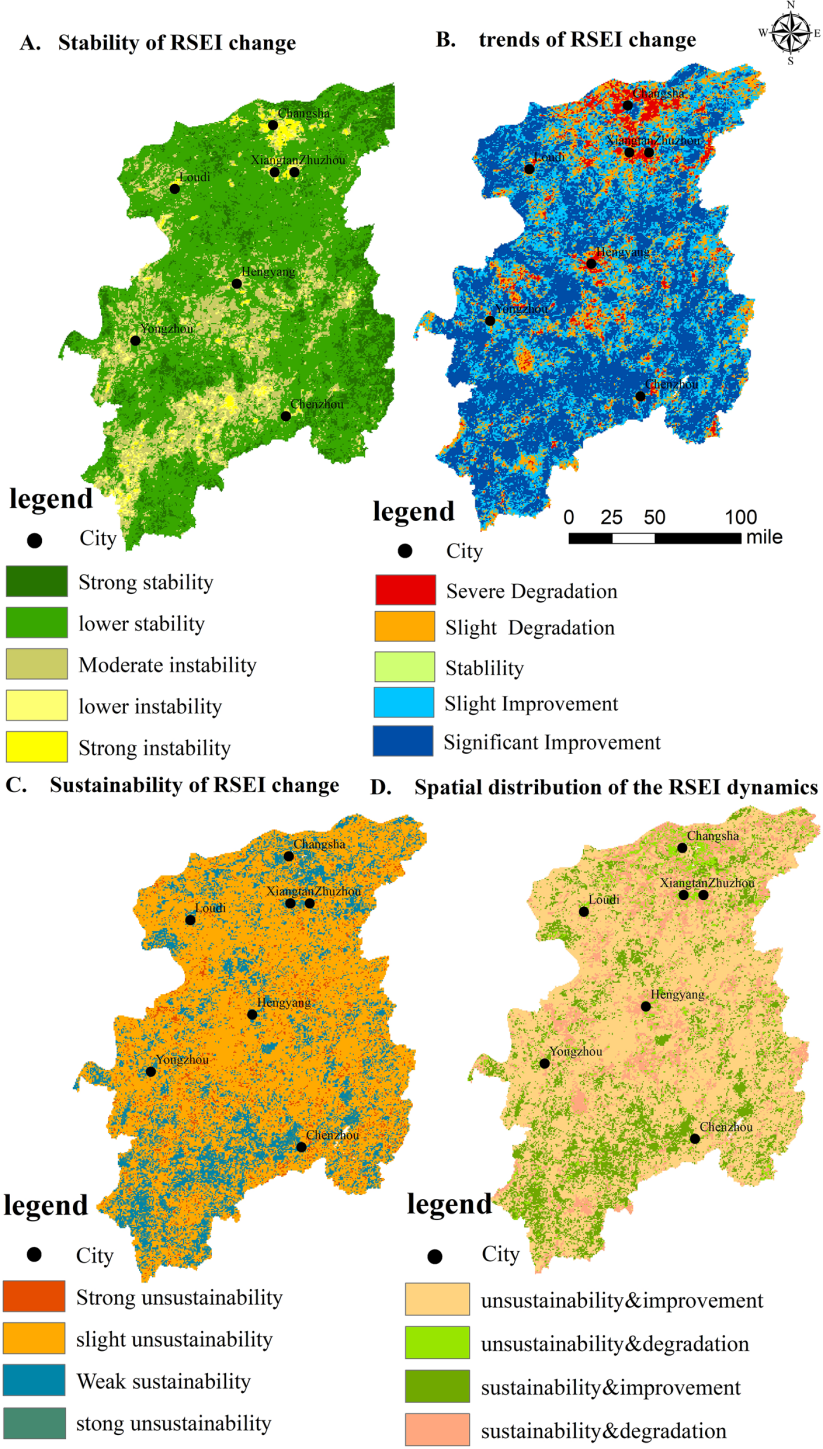
(**a**) Spatial distribution of the coefficient of variation of the interannual RSEI in the Xiangjiang Basin from 2001 to 2020, (**b**) trends of interannual RSEI change in the Xiangjiang Basin from 2001 to 2020, (**c**) sustainability of interannual RSEI change in the Xiangjiang Basin from 2001 to 2020, (**d**) spatial distribution of the RSEI dynamics based on trends and the Hurst index.

According to spatial distribution characteristics, areas with poor ecological quality and those with large fluctuations in the Xiangjiang River Basin are closely related to the distribution of cities. The areas with poor ecological quality in the Xiangjiang River Basin are mainly concentrated in Changsha, Zhuzhou, Xiangtan and Hengyang city centers. Using the CV coefficient of variation to obtain the distribution map of RSEI’s change intensity over the past 20 years (Fig. 3a), the areas with large ecological environment fluctuations are mainly located in the center of Changsha City, the center of Xiangtan, Hengyang and most county centers. Consistent with areas with poor ecological environments, the land use types in areas with large ecological environment fluctuations are mainly construction land. Thus, human activities negatively impact regional ecological quality and also reduce the stability of ecological quality. The spatial distribution, the range of RSEI values, and the trend of changes correspond with the results of the previous study on Chang-Zhu-Tan using Landsat images^52^, reducing the differences caused by the selection of various remote sensing images.

### Analysis of the trend of ecological environment change

Figure 3 shows the results obtained using the method of Sen-Mk to study the changes in the ecological environment (where |Z|> 1.96, α > 95% means significant change and |Z|< 1.96, α > 95% means no significant change). The overall changes in the Xiangjiang River Basin are mainly concentrated in slight and significant improvements, accounting for 32.51% and 47.51% respectively. Moreover, the proportion of degraded and unchanged is relatively small; only 0.12% is unchanged, while 14.95% and 5.01% are slightly and severely degraded, respectively. From the spatial distribution, the spatial distribution of areas with severe ecological quality degradation was relatively concentrated. They are mainly located in the middle and lower reaches, and the lower reaches are the most serious.

The sustainability of the ecological quality of the Xiangjiang River Basin was analyzed using the Hurst index to explore the possible spatial risk areas of the ecological quality. The results are shown in Fig. 3c. The range of the index was between 0.128 and 0.900. Based on the original classification of the Hurst index, the Hurst index was further classified, and the classification results are shown in Table 4. Areas with a grid value of 0.5 in the Hurst index of the Xiangjiang River Basin are unavailable; that is, there is no area with uncertain directions. The data obey a normal distribution. Thus, the Xiangjiang River Basin is dominated by weak persistence. Furthermore, the Xiangjiang River Basin is generally dominated by unsustainability. The areas with strong and weak unsustainability accounted for 77.79%, while the areas with sustainability accounted for less than 13%. The regional distribution of unsustainability was relatively scattered, while the area of positive persistence was highly concentrated. Positive persistence is mainly distributed upstream and downstream, such as in Daoxian County, Ningyuan County, Jiahe County, Guiyang. County, and Beihu District. Moreover, the upper reaches are mainly distributed in the Yuelu District, Yuhua District, Western Changsha County and Yuhua District.

**Table 4:** Classification of Hurst index.

Value	Type
0 ≤ hurst < 0.3	Strong unsustainability
0.3 < hurst < 0.5	Weak unsustainability
Hurst = 0.5	Uncertain
0.5 < hurst < 0.7	Weak sustainability
0.7 < hurst < 1	Strong sustainability

The results of the Theil–Sen Median slope estimation obtained in the previous section (degradation and improvement of ecological environment) and the Hurst index obtained in the previous section (positive persistence and anti-sustainability) are utilized to effectively visualize the development trend of the ecological environment in the Xiangjiang River Basin. Furthermore, the analysis results are merged to obtain the changing trend of the Xiangjiang River Basin(Fig. 3d), which can be divided into four categories: positive continuous improvement, reverse continuous improvement, positive continuous degradation, and reverse continuous degradation. The total area of continuous degradation is approximately 3189 km^2^, accounting for 4.52% of the total area. The total area of positive continuous degradation is approximately 13,077 km^2^, accounting for 15.46% of the watershed’s total area. The total area of reverse continuous improvement is approximately 52,689 km^2^. Among the four types of areas the proportion is the highest, at approximately 62.31%, and the total area of positive continuous improvement is approximately 14,977 km^2^, accounting for approximately 17.71% of the total area of the watershed. For future developments, areas with anti-sustainable improvement and positive continuous degradation will be the future ecological environment. Thus, deterioration is possible. From the data analysis, this part of the area accounts for 77.77% of the total area of the Xiangjiang River Basin. The part that is continuously degrading is mainly distributed in the periphery of Changsha and Xiangtan city centers. With the gradual acceleration of urbanization, cities continue to expand to their peripheries and suburbs, and the ecological quality of the suburbs is easily threatened. Therefore, considering the future of the Xiangjiang River Basin, the hidden danger of ecological environmental degradation is relatively large. During future ecological protection, the red (positive and continuous degradation) and orange (anti-sustainable improvement) parts must be emphasized to achieve maximum economic and ecological sustainability.

### Driving force analysis

**Figure 4:**
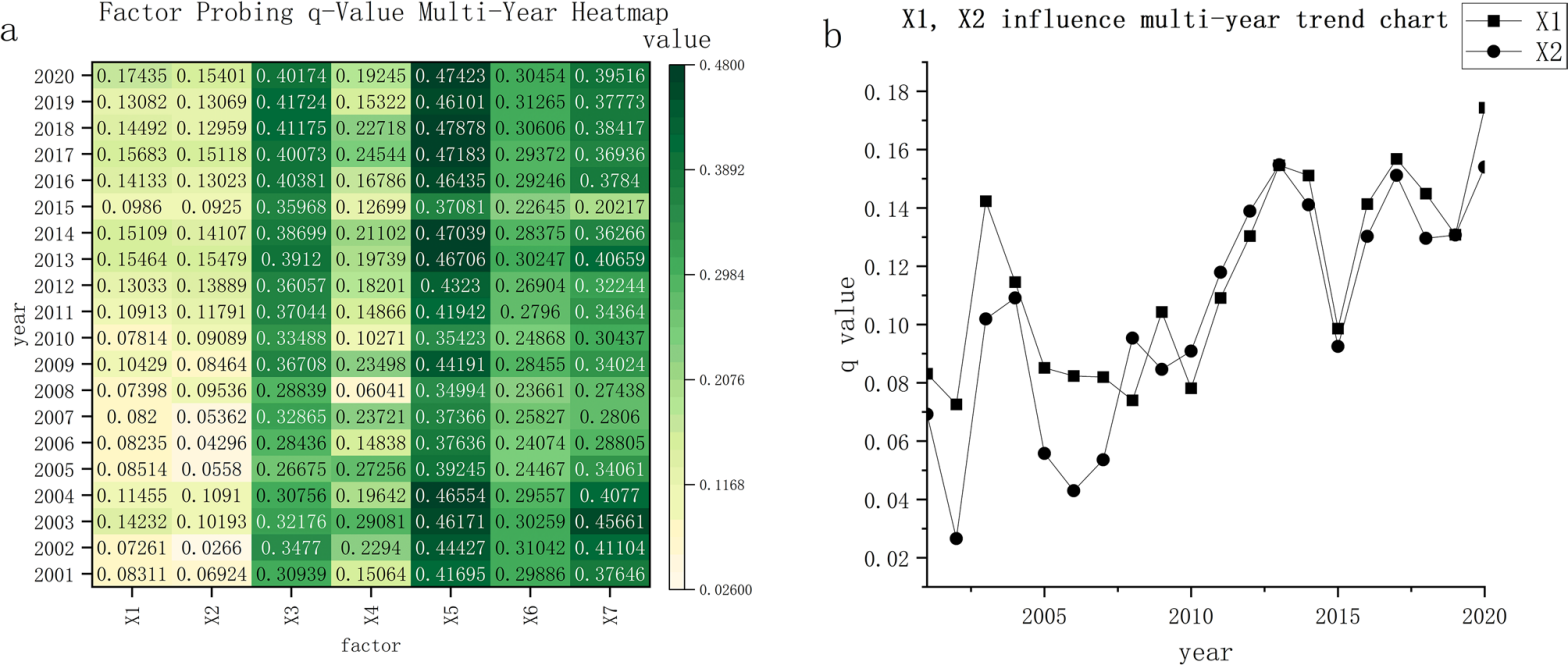
(**a**) Geodetector results heatmap (p-values all < 0.05), (**b**) trend chart of the influence of X1 and X2 over the years.

Seven independent variables were detected as factors. Based on the geographic detector. Figure 4a shows that human factors playing major roles in ecological change mainly include land use (X3), wherein the explanatory power can be up to 41.7%, and population density (X5), wherein the explanatory power can reach up to 47.8%. Furthermore, the natural factors are terrain (X7) and slope (X6), which have explanatory power reaching 45.6% and 31.2%, respectively. RSEI is obtained from four indicators through PCA, and every single indicator is affected by several factors. Therefore, the level of explanatory power can explain the influencing factors of the ecological environment. From a longitudinal perspective, GDP (X2) and distance from the city center (X1) have relatively lower explanatory power, at only approximately 10%. However, over time, the explanatory power of GDP shows an upward trend yearly (Fig. 4b), from 8% in 2001 to 17% in 2020. The distance from the center also increased from 6% in 2001 to 15% in 2020. The explanatory power of land use rose from 30.9% in 2001 to 40.1% in 2020, and that of population density has remained at 47% since 2016. The importance of human activities to the ecological environment continues to increase with the acceleration of the urbanization process, which corresponds with many research results^53,54^ and explains the factors to a degree. In addition, the accuracy of the factor detection results was verified.

**Figure 5:**
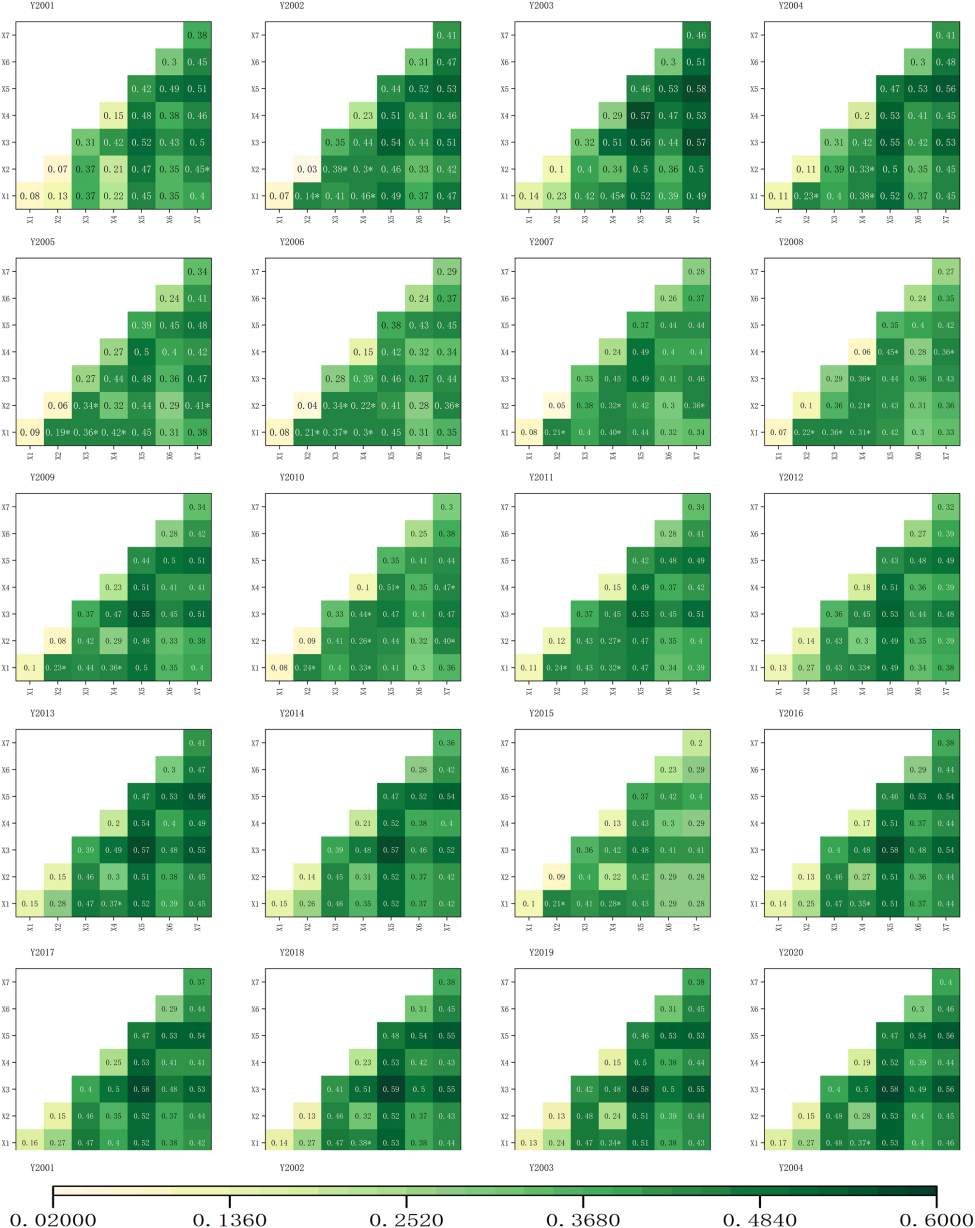
Interaction Results Plot (where with * is a two-factor nonlinear enhancement, without * is linear enhancement).

The interaction detector was used to study the interaction of the influencing factors in the study area and to examine the effect of multiple factors on regional ecological quality. The results shown in Fig. 5 show that the q value in the interaction of any two factors is substantially larger than that of a single factor, the interaction of X1–X7 is all linear or nonlinear enhancement, and linear enhancement is the primary; that is, the superposition of the two factors has played a powerful role in promoting RSEI. Considering the interaction effect from the mean value of each year, the most powerful human factor is still X5 (population density), and the most influential natural factor is X7 (slope). When comparing the two, human factors play a leading role in ecological quality changes. The human population should be balanced to promote the sustainable development of the ecological environment in the Xiangjiang River Basin. The relationship with nature aims to achieve a harmonious coexistence between man and nature. However, with the recent development of the economy, population density has continued to increase, and soil erosion has continued to intensify. Hunan Province has become one of the provinces with the most severe soil erosion in the Yangtze River Basin^55^. Particularly, in the hilly areas of the Xiangjiang River Basin, the terrain is broken, the precipitation is concentrated and is mostly heavy rain, the area of easily eroded soil and parent rock is large, and the soil erosion is extensive. The slope is one of the most important factors affecting soil erosion. Therefore, ecological quality is affected by the slope.

## Discussion

### Spatiotemporal distribution characteristics of the remote-sensing-based ecological index

RSEI has good stability and is widely used in the ecological quality evaluation of different landscapes. The RSEI values of various landscapes are different: approximately 0.43–0.54 in plateau areas^56^, approximately 0.24 in desert areas^57^, approximately 0.49–0.69 in large river floodplains or watershed areas^58^, and approximately 0.63 in densely vegetated areas^59^. The mining and urban areas are approximately 0.4–0.5^60^, and 0.4–0.7^61^, respectively. The RSEI in this paper is between 0.589 and 0.718, which corresponds to the numerical range of cities and watersheds. The CV coefficient and Sen trend test methods revealed that the areas with large ecological environment fluctuation values and those with serious ecological degradation were concentrated near the city or county centers. This condition is due to the dense and industrially developed population of the Chang-Zhu-Tan urban agglomeration. In addition, this area is a concentration of leading industries in Hunan Province, such as iron and steel, petrochemicals, energy, and construction machinery. The total amount of industrial sewage discharge is relatively large, and the number of pollutants and amount of artificial heat are larger and denser than those in other places^19,62^. Combined with the dense buildings in this area, the dryness value is higher than in other places, and the vegetation coverage is lower than in other places^4^, resulting in low ecosystem service value and poor ecological quality^18^. In particular, Furong District and the most central part of Hengyang are in significantly improved space when the surrounding areas are degraded. Furong District was already undergoing construction in 2001. After 20 years of development, the construction has Continued to expand to the outer suburbs, and some polluting enterprises have also begun to migrate to the periphery, while the urban center has improved its ecological quality with measures such as artificial urban greening, and is also closely related to the Juzizhou scenic spot. The phenomenon indicating the improvement in the ecological environment of the city center is also reflected in previous studies. For example, Esau Igor et al.^63^ found that in cities (ranging from 5 to 10 km near the city), vegetation has deteriorated because of urbanization, while the vegetation coverage in the old urban area has increased. Simultaneously, Kumpula et al.^64^ also indicated that the vegetation type of the old urban area has changed from a single functional biological community to a complex biological community due to the intervention of human activities, which will also tend to improve the NDVI of the old city. NDVI is one of the important indicators affecting RSEI. Therefore,it can explain why the Furong District and Hengyang City Center are areas with improved ecological environments.

### Factors influencing of temporal and spatial variations of the remote-sensing-based ecological index

The results of factor detection show that the natural environment also limits the scope of human activities to a large extent, affecting the quality of the ecological environment. The spatial differentiation of RSEI in the Xiangjiang River Basin from 2001 to 2020 was not controlled by a single factor, because each factor affecting the temporal and spatial differentiations of RSEI also had spatial differences or changed with time. Precipitation has a reduced effect on RSEI than topography and slope, possibly because of the location of the Xiangjiang River Basin in the subtropical monsoon climate region, with abundant rainfall and a dense river network in the basin. Therefore, vegetation growth is unaffected by water stress, and the interference on RSEI is small, as reflected in previous studies. For example, Sun Zhijuan et al. studied the relationship between vegetation NPP and annual precipitation in Yunnan Province and found that the relationship between precipitation and vegetation NPP was small^54^. Li et al.^65^ applied methods such as linear trend regression analysis Hurst index analysis, and geodetector models to explore the spatial and temporal variation characteristics and drivers of vegetation cover in the southwest trough region, and found that the relationship between precipitation and vegetation growth was small.

**Figure 6:**
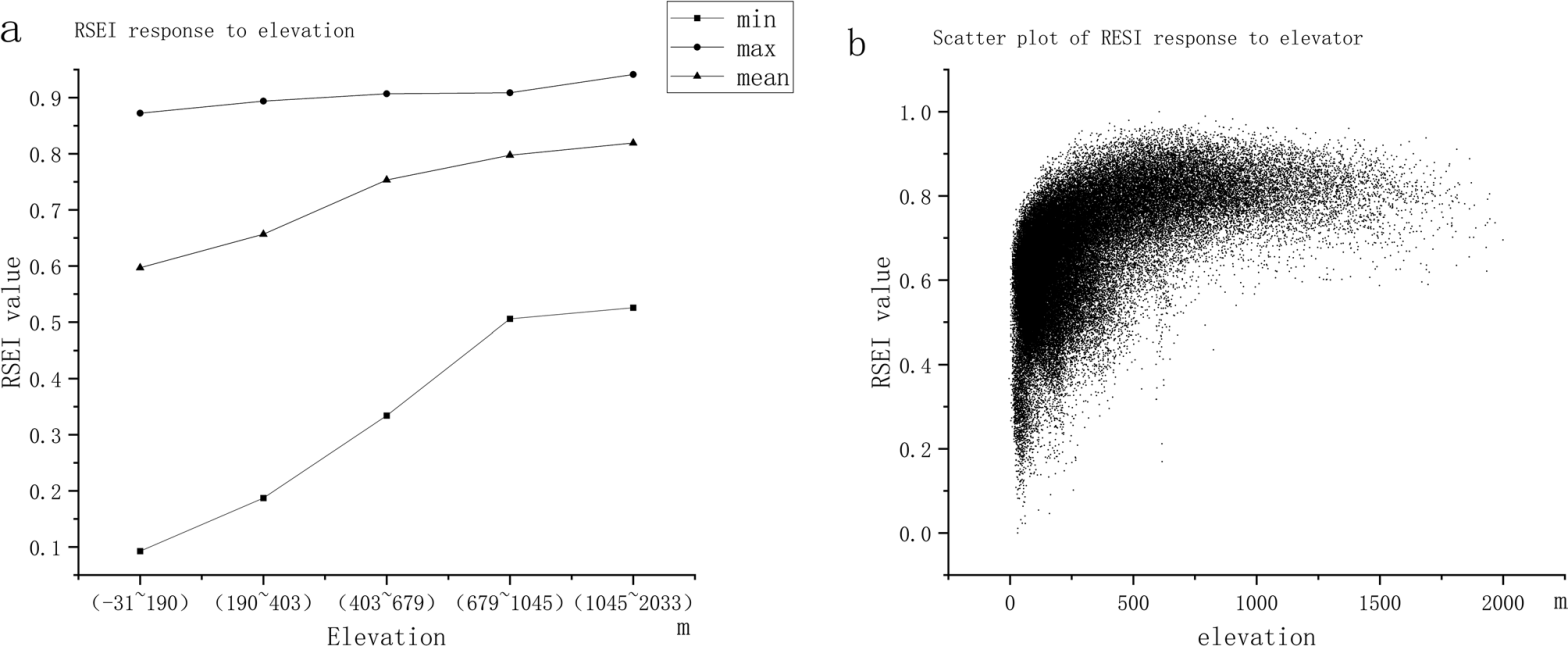
(**a**) RSEI values for different altitude ranges, (**b**) scatter plot of the relationship between RSEI and altitude.

Terrain and slope can be considered constant factors on a 20-year scale. Thus, the RSEI data for 2020 were used to study the effects of the two factors above on the spatial heterogeneity of RSEI. The results shown in Fig. 6, show that a high RSEI value leads to improved ecological quality, with the increase in altitude. This finding indicates that altitude and ecological quality in the Xiangjiang River Basin are positively correlated. Human activities are intensive at lower altitudes, indicating that the decline in the ecological quality of the Xiangjiang River Basin is closely related to human activities. Elevation affects factors, such as water-heat combination and soil type, resulting in different vegetation habitats and types in various altitude ranges of the Xiangjiang River Basin, and forming vertical zonal differentiation of vegetation in the mountains. Different altitude ranges also constrain the intensity and type of human activities,, resulting in differences in the vertical distribution patterns of NDVI, humidity, and dryness, thus affecting RSEI. This finding corresponds with previous research results on the response of RSEI to elevation in the Yangtze River Basin^66^.

**Figure 7:**
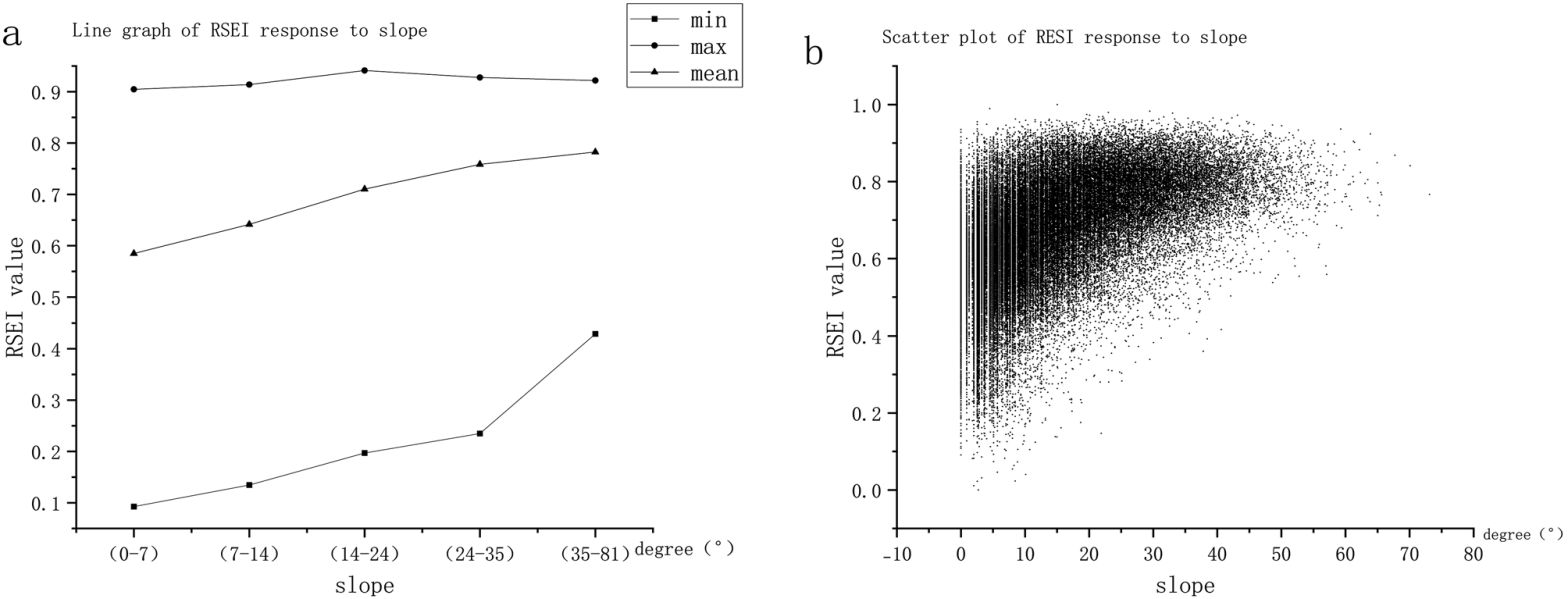
(**a**) Values of RSEI in different slope ranges, (**b**) scatter plot of the relationship between RSEI and slope.

The research results of the slope (Fig. 7) show that as the slope becomes steep, the RSEI value increases, and the ecological quality continues to improve, indicating a positive correlation between the slope and the ecological quality. Places with steep slope is not conducive to people’s residence and life. Therefore, human activities have minimal interference in places with large slopes, and the ecological quality is superior, which further shows that the ecological quality of the Xiangjiang River Basin is closely related to human activities. Several scholars have found a relationship between NDVI and slope in previous studies. Liu et al.^67^ and Deng et al.^68^ found through their studies that the 0° to 5° area is the most important occurrence area of vegetation degradation. This area is where human agricultural activities, industrial activities, and town construction are conducted, and vegetation is encroached upon by human activities. With the gradual increase in slope, the vegetation is slightly affected by the intervention of human activities, and vegetation degradation is mostly affected by natural disasters, which corresponds with the findings of this paper. In addition, Zhang et al.^69^ argued in their study that vegetation restoration should first consider the slope factor, and one of the important indexes of RSEI is NDVI, which accounts for additional weight in the PCA. Therefore, assuming that ecological quality is related to the slope is reasonable.

**Figure 8:**
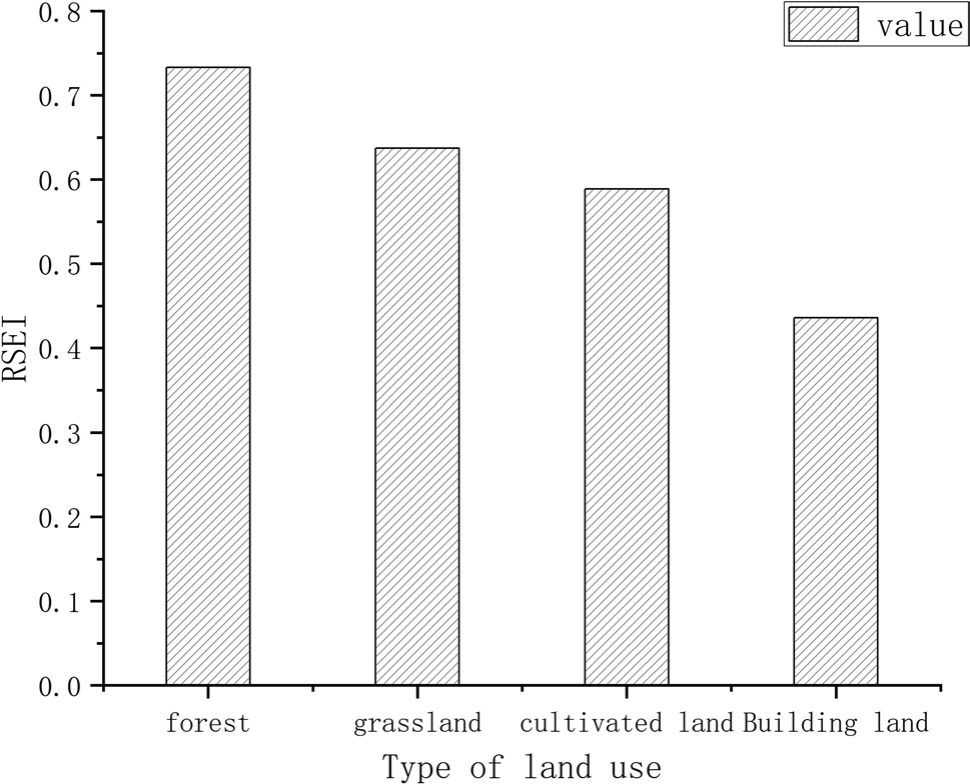
Relationship between RSEI and land use.

Direct factors, such as land use/cover change, directly contribute to ecological quality. In particularly, vegetation restoration can prevent soil erosion, improving ecological quality^26^. Therefore, changes in land use/cover types are direct and key factors affecting ecological quality. Previous studies have found that land use/cover types are positively correlated with land use/cover types. Moreover, they showed that high ecological quality, was negatively correlated with those with low ecological quality. According to the research results of this paper (Fig. 8), the average RSEI value of construction land is 0.436, the average RSEI value of grassland is 0.637, the average RSEI value of cultivated land is 0.589, and the average RSEI value of forest land is 0.733. The results of the research and the geographic detector are consistent, indicating that from only the ecological viewpoint, the ecological value of different land use methods is: forest land > grassland > cultivated land > construction land, and the mean value of RSEI under different land use methods is remarkably different. Hence, changing the current status or changing trends of direct influencing factors such as land use/cover type, ultimately affects ecological quality^46,47^.

These conclusions are reflected in regional studies related to Hunan Province or the Xiangjiang River Basin. For example, Zheng et al.^70^ used the land use transition matrix and the investment model to evaluate the ecological quality of Nanyue District (within the Xiangjiang River Basin). The results show that the expansion of construction land will induce a decline in the quality of the ecological environment, which is closely related to land use patterns. Peng et al.^71^ concluded in their research on ecosystem services in Changsha that land use changes caused by rapid urban expansion will induce a continuous decline in ESV, slowing down the downward trend of ESV. Wang et al.^71^ evaluated the supply-and-demand relationship of ecosystem services in Hunan Province. The results show that the spatial distribution of areas with high demand and low supply highly corresponds with the ecologically degraded areas in this paper, which is consistent with the area of ecological improvement. The above studies are corresponds with the conclusions of this paper.

Notably, severe anthropogenic disturbances, such as high population density and GDP, have introduced enormous pressure on ecosystem health, resulting in a decline in ecological quality^26^. This study also reached similar conclusions. Regardless of the spatial distribution of RSEI or the long-term change trend, poor ecological environment quality, degradation, or large fluctuations are all indicated in the Chang-Zhu-Tan urban agglomeration, the center of Hengyang City, sporadic distribution in the center of each city or county. and other economically developed areas. From 2001 to 2020, the distance between the city center and GDP has become increasingly powerful in explaining RSEI, which fully reflects the interference effect of human activities and economic development on ecological quality. The Hunan government proposed the construction goal of "3 + 5" urban agglomeration, focusing on building three cities of Changsha, Zhuzhou and Tan, and five cities of Yueyang, Changde, Yiyang, Loudi and Hengyang. The goal was to drive the rapid economic development of Hunan with the “3 + 5” urban agglomeration as the main body and promote the rapid urbanization of these areas, turning the original forest land into non-forest land, thereby reducing the ecological quality of these areas. In 2011, the whole country began to implement the construction of an ecological civilization to improve ecological quality on a large scale. Hence, Hunan Province proposed a series of measures to protect the environment. For example, the "Hunan Province Xiangjiang River Protection Regulations" promulgated in 2012 further improved the policy support for ecological environmental protection in the Xiangjiang River Basin. In 2013, Hunan Province promulgated three consecutive "three-year action plans" to realize the policy of "clear water, green water on both sides, and beautiful urban and rural areas" in the Xiangjiang River Basin. A 3-year protection policy has been implemented. Furthermore, coupled with the introduction of the main functional zoning in Hunan Province, the ecological environment of some restricted development zones has gradually improved. Therefore, the spatial heterogeneity of the ecological environment in the Xiangjiang River Basin is relatively strong;

### Innovation and shortage

Many studies on ecological quality in Hunan Province or the Xiangjiang River Basin are available, but studies on the trends of changes in long time series and the influencing factors are limited. Yuan et al.^26^ used correlation to study the influencing factors that substantially change in ecological quality in the Dongting Lake basin. Based on the previous studies, geographic probes to further screen the possible influencing factors and their effects on the temporal and spatial changes of ecological quality in the Xiangjiang River Basin, which compensated for the lack of research in this area, provided ideas for the subsequent ecological protection of the Xiangjiang River Basin and provided a driving force for further ecological changes. As an important development area in Hunan Province, the Xiangjiang River Basin should take on new responsibilities and innovative wisdom in ecological environmental monitoring and management. The comprehensive use of remote sensing technology and the GEE platform provide a good means to conduct in-depth monitoring of the urban ecological environment in Hunan Province and realize the harmonious coexistence between humans and nature.

Regrettably, only the total economic volume is considered, and the ecological impact of each industry is disregarded in this paper. Furthermore, this paper uses MNDWI to mask out the water body part of the Xiangjiang River Basin to avoid the influence of large-scale waters in the PCA of the indicators. Thus, this paper focuses more on evaluating the ecological quality of non-water parts. However, the water in the Xiangjiang River Basin, which flows into the Yangtze River through Dongting Lake, plays an important role in maintaining the ecological quality and safety of the Xiangjiang River Basin, Dongting Lake and the Yangtze River. Future research could use a comprehensive evaluation which is an effective evaluation method or ecological quality to study the changes in the Xiangjiang River water body and provide additional targeted scientific data support for the protection and management of the Xiangjiang River Basin.

## Conclusion

In this study, we use GEE online processing platform to process remote sensing images and four indicators were used to construct the RSEI of the Xiangjiang River Basin. Moreover, the temporal and spatial evolution laws and drivers in the Xiangjiang River Basin were explored. The conclusions are presented as follows.

1. The change trend of RSEI showed a slow increase or a slight decrease before 2011, and a rapid increase after 2011, indicating that the overall ecological quality of the Xiangjiang River Basin has continued to rise over the years. Further, the ecological quality of the center of the Chang-Zhu-Tan urban agglomeration, Hengyang, continues to decline, with high volatility and poor ecological and environmental stability.
2. The sustainability results show that the suburbs of Changsha and Hengyang are in a state of anti-sustainable improvement, indicating degradation risk to its ecological environment with expanding urbanization.
3. The geographic detector results show that the order of impact on the ecological environment from small to large is population density > elevation > land use pattern > slope > precipitation > distance from the city center > GDP; The distance from the city center and the influence of GDP continue to increase year by year, and the interaction results show that land use pattern and population density, slope and density have the strongest interaction. Land use has an intuitive impact on ecological quality. The ecological quality of forest land is the best, followed by cultivated land, and the quality of construction land is the worst. Simultaneously, ecological quality gradually improves with increasing altitude and slope.

## Supplementary Information

Supplementary Information.

## Data Availability

The MODIS iamge that support the findings of this study are openly available in GEE (https://earthengine.google.com/). The data used is from the NASA website (https://urs.earthdata.nasa.gov/). Land use data from the paper "30 m annual land cover and its dynamics in China from 1990 to 2019" (ESSD-The 30 m annual land cover dataset and its dynamics in China from 1990 to 2019 (copernicus.org). The data of GDP come from China County Statistical Yearbook (www.stats.gov.cn/tjsj./ndsj/). The data of precipitation come from National Climatic Data Center (https://data.cma.cn/). The data of population density come from the WorldPop (https://hub.worldpop.org/). The data of downtown data come from the National Bureau of Statistics of China (http://www.stats.gov.cn/).
