# Supplementary material for: Analysis of ecological quality changes and influencing factors in Xiangjiang River Basin
Source: Sci Rep. 2023 Mar 16;13:4375. doi: 10.1038/s41598-023-31453-7 (PMC10020476; doi:10.1038/s41598-023-31453-7)
Supplement: Supplementary file 1 — Supplementary Information. [file 41598_2023_31453_MOESM1_ESM.pdf]

Figure S1: Spatial distribution of RSEI in the Xiangjiang Basin between 2001 and 2020

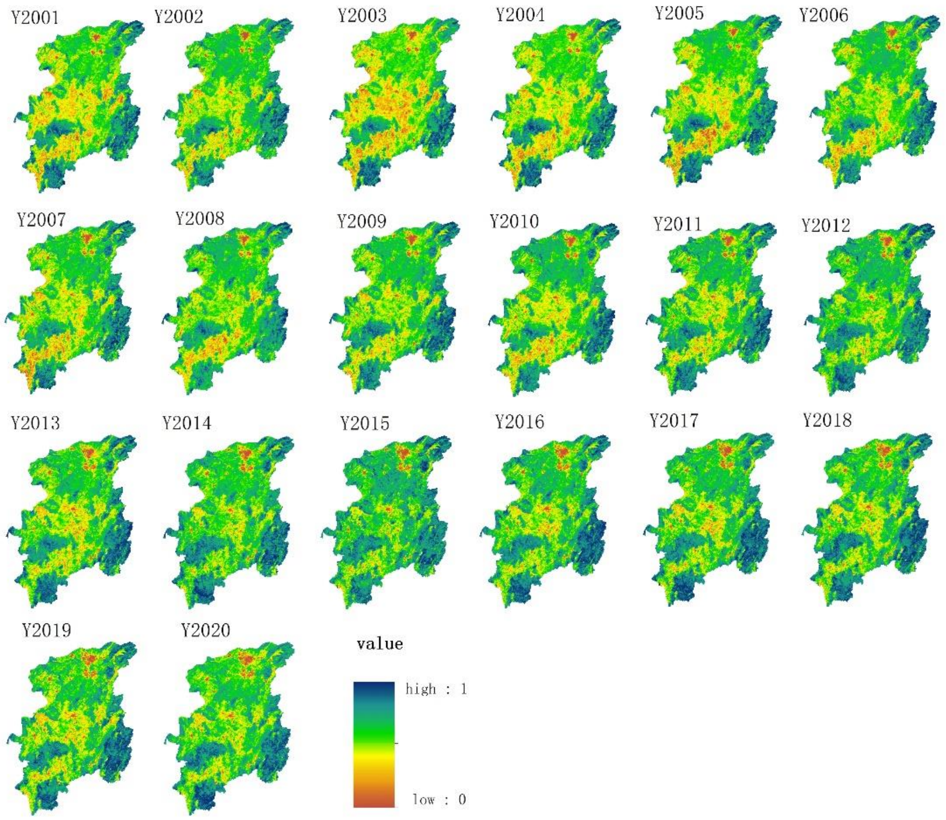

Appendices1: analysis results of RSEI principal component from 2001 to 2020 (PCA2)

index	NDVI	LST	WET	NDBSI	EV(PC2)	ECR(PC2%)
2001	-0.379	0.302	0.812	-0.323	0.007	16.4
2002	0.712	0.644	-0.224	-0.163	0.007	21.6
2003	0.281	0.757	0.323	-0.492	0.005	11.2
2004	0.745	0.352	-0.561	-0.068	0.006	14.5
2005	0.766	0.544	-0.172	-0.293	0.008	20.1
2006	0.674	0.633	-0.233	-0.298	0.007	18.8
2007	0.764	0.432	-0.468	-0.093	0.006	14.3
2008	0.721	0.505	-0.391	-0.269	0.007	18.7
2009	0.675	0.583	-0.404	-0.197	0.007	15.7
2010	0.722	0.553	-0.382	-0.157	0.007	16.4
2011	0.652	0.654	-0.327	-0.197	0.006	15.8
2012	0.655	0.622	-0.351	-0.244	0.007	19.3
2013	0.644	0.643	-0.301	-0.281	0.007	14.8
2014	0.713	0.599	-0.358	-0.058	0.007	20.1
2015	0.717	0.428	-0.538	0.106	0.006	18.8
2016	0.664	0.327	-0.671	0.037	0.005	13.3

2017	0.702	0.603	-0.342	-0.157	0.006	14.1
2018	0.688	0.581	-0.432	-0.092	0.005	13.6
2019	0.711	0.575	-0.396	-0.068	0.006	13.5
2020	0.730	0.663	-0.096	-0.133	0.008	17.8

Appendices2: analysis results of RSEI principal component from 2001 to 2020 (PCA3)

index	NDVI	LST	WET	NDBSI	EV(PC3)	ECR(PC3%)
2001	0.609	0.689	-0.121	-0.372	0.006	14.1
2002	-0.272	0.495	0.771	-0.292	0.006	17.7
2003	0.691	0.092	-0.713	0.069	0.005	10.4
2004	0.0311	0.699	0.537	-0.471	0.005	12.6
2005	-0.334	0.534	0.714	-0.303	0.004	11.1
2006	-0.286	0.478	0.791	-0.251	0.006	14.5
2007	-0.053	0.719	0.633	-0.278	0.004	10.6
2008	-0.163	0.575	0.705	-0.381	0.005	14.3
2009	-0.211	0.579	0.679	-0.397	0.005	11.1
2010	-0.149	0.597	0.716	-0.328	0.004	10.4
2011	-0.241	0.507	0.747	-0.353	0.004	9.18
2012	-0.229	0.499	0.735	-0.397	0.004	10.6
2013	-0.242	0.447	0.778	-0.366	0.004	8.81
2014	-0.137	0.562	0.726	-0.371	0.004	10.8
2015	0.024	0.767	0.598	-0.228	0.003	10.7
2016	0.144	0.772	0.500	-0.363	0.004	10.8
2017	-0.185	0.536	0.735	-0.369	0.004	9.63
2018	-0.145	0.622	0.689	-0.341	0.003	8.15
2019	-0.138	0.625	0.709	-0.294	0.004	9.35
2020	-0.357	0.438	0.758	-0.324	0.004	8.51

Appendices3: analysis results of RSEI principal component from 2001 to 2020 (PCA4)

index	NDVI	LST	WET	NDBSI	EV(PC4)	ECR(PC4%)
2001	-0.544	0.037	-0.527	-0.651	0.0009	2.09
2002	-0.438	0.177	-0.538	-0.667	0.001	3.76
2003	-0.513	0.007	-0.561	-0.649	0.001	2.07
2004	-0.476	-0.008	-0.556	-0.681	0.0008	2.12
2005	0.451	-0.088	0.567	0.682	0.001	2.74
2006	-0.565	0.113	-0.482	-0.659	0.001	2.86
2007	-0.507	0.178	-0.533	-0.653	0.0009	2.33
2008	-0.548	0.035	-0.512	-0.659	0.001	2.86
2009	-0.541	0.027	-0.558	-0.627	0.0008	1.83
2010	-0.519	0.129	-0.521	-0.664	0.001	2.56
2011	-0.548	0.086	-0.536	-0.635	0.0007	1.78
2012	0.548	-0.024	0.534	0.642	0.0008	2.15
2013	-0.559	0.037	-0.504	-0.656	0.0006	1.22
2014	0.471	-0.162	0.555	0.666	0.0009	2.33
2015	0.461	-0.246	0.546	0.654	0.001	3.38
2016	-0.528	0.111	-0.507	-0.671	0.001	2.29

2017	-0.495	0.095	-0.534	-0.678	0.0009	2.28
2018	-0.496	0.095	-0.528	-0.528	0.0006	1.64
2019	-0.485	0.161	-0.521	-0.683	0.001	2.07
2020	0.386	-0.203	0.590	0.678	0.001	2.45
